# Saturnine Encephalopathy

**Published:** 1907-09-21

**Authors:** 


### Illustrative Cases.

### SATURNINE ENCEPHALOPATHY.

Lead poisoning will produce cerebral disturbance and epileptiform convulsions associated with coma, either as well as, or independently of, its other toxic effects. It now and then happens that a patient is brought in comatose, having been found lying unconscious with a gash upon his head, and the diagnosis may for the time being be very obscure. The first point to settle, if possible, is whether or not the head injury caused the unconsciousness, or whether the unconsciousness came first, the head being injured in falling. From the point of view of immediate treatment the thing to decide is (1) whether to operate upon the cranium, for the relief of depressed fracture, or meningeal hæmorrhage; or (2) whether to wash the stomach out to remove any poison, such as laudanum, that may have been taken; or (3) whether to leave the patient absolutely still lest he should have a cerebral hæmorrhage, which might otherwise be increased.

We do not propose to discuss all the points in the differential diagnosis of cases of coma here. We only wish to lay stress upon the fact that lead-poisoning is a possible cause, so that in every case of coma the gums should be looked at to be sure that no blue line is there. It takes almost no time to look at the gums, and occasionally they may greatly help the diagnosis.

### An Illustrative Case.

The following is an example of saturnine encephalopathy arising whilst the patient was already under observation for other symptoms of lead-poisoning. The case is that of a man, aged 27, a painter, who lived and worked in a small country town. He was employed at house-painting for nearly seventeen years, and had colic six or seven times, the first occasion being ten years ago. His attacks were generally attended with severe griping pain about the umbilicus, associated with constipation, both being readily overcome by a dose of castor oil. Eight years ago he experienced slight weakness of the hands, but he recovered perfectly. He now sought advice for vomiting, cramps in the legs, severe griping abdominal pain, and constipation. These increased in severity, so that he was confined to bed entirely. Three weeks later he noticed his hands becoming weaker, and after a day or two he had pains in the shoulders and down the arms. He was fairly nourished, but the muscles of the forearms, both flexors and extensors, became wasted. There was a well-marked blue line on the gums. The lungs and heart were normal, the abdomen rather contracted and somewhat tender. The urine had a specific gravity of 1017; it was free from albumen and sugar, and did not contain enough lead to give a visible cloud with sulphuric acid. The colic became very severe; it was not relieved by castor oil, but croton oil with opium, given internally, and belladonna and chloroform liniment, applied to the abdomen externally, allayed it. Iodide of potassium was given as his routine medicine.

A few days later he became very restless and semi-delirious, gradually sinking into a coma, in which there was involuntary evacuation of urine and fæces. The pulse was now 160, weak and fluttering; the pupils were natural and reacted to light. He remained continuously comatose for three days, on the second of which in the early morning he had a short epileptiform convulsion, with foaming mouth and clenched hands. Some urine drawn off by catheter immediately afterwards had a specific gravity of 1014, and contained both albumen and a body which reduced Fehling's solution; microscopically no renal tube casts were found. The day after the fit he became semi-conscious again, the bowels having been freely opened by compound elaterium powder; the pulse was now 94; the patient understood some things that were said to him, but he did not himself speak. There was no paralysis of limb or cranial nerve. Next day he was quite sensible; the pulse was 100; the urine still contained a little albumen and a trace of some reducing substance. The latter was gone next day, and the albumen disappeared a little later. The patient slowly convalesced; he had no recurrence of cerebral symptoms; the paresis of his arms lasted longer than the rest of his troubles.

### Points in Diagnosis.

There could be little doubt that the above was an instance of saturnine encephalopathy. If the coma had come on when he was at work the diagnosis would have been very obscure unless the gums had been looked at. Alternative diagnoses might be (1) uræmia, (2) true epilepsy, (3) cerebral hæmorrhage; but there are strong arguments against these. There was no albuminuria either before or after the coma, there was no big heart, nor did the urine contain tube casts, so that although lead may cause granular kidney, and therefore either uræmia or cerebral hæmorrhage, there was no evidence at all that this patient had granular kidney. The fact that the coma came on long before the fit, and the fact that there was only one fit during three days' coma, are arguments against epilepsy and uræmia respectively. There was no evidence of any paresis other than that due to the peripheral neuritis, so that cerebral hœmorrhage in its usual position was excluded, besides which the age of the patient, and the general course of the coma, convulsion, and subsequent speedy recovery, are against it.

Convulsions and coma are the commonest symptoms of saturnine encephalopathy; if there is one point in which the above case is not typical of the majority it is that there was but a single epileptiform convulsion during the long period of coma, instead of a dozen, twenty, or even fifty or more. The fact that the patient recovered completely after being quite desperately ill is according to the rule in this condition.

